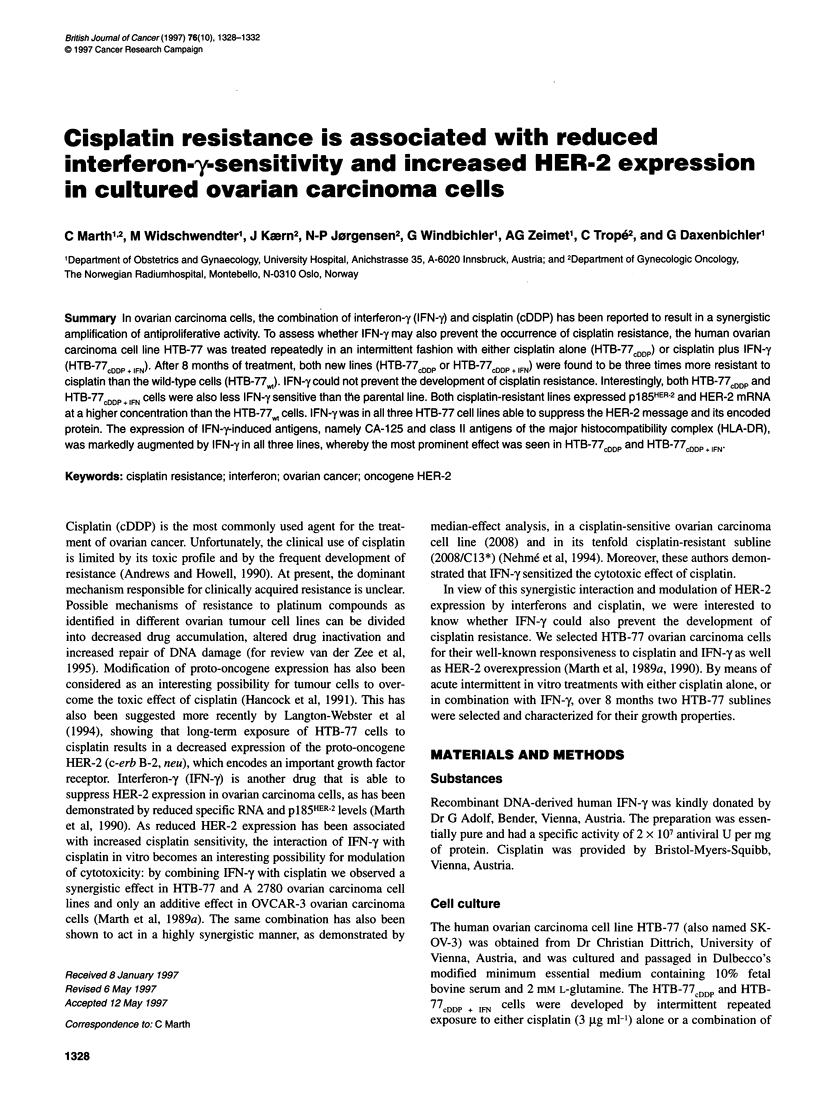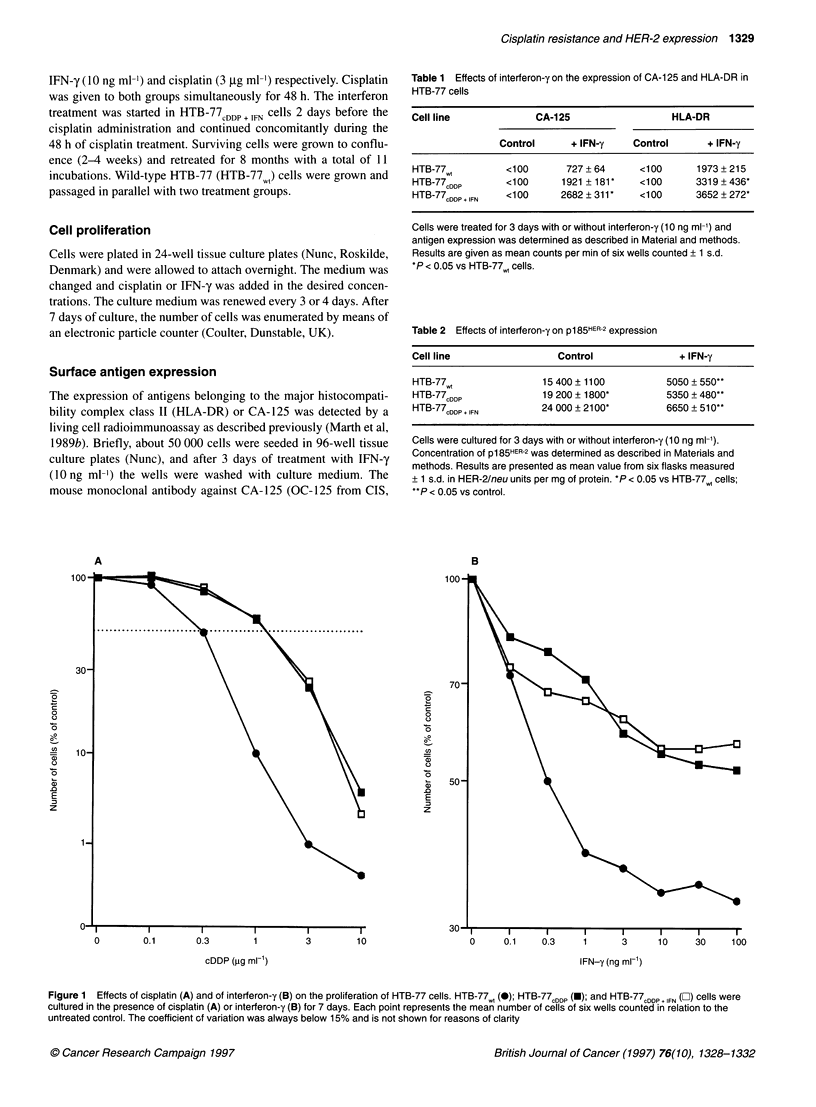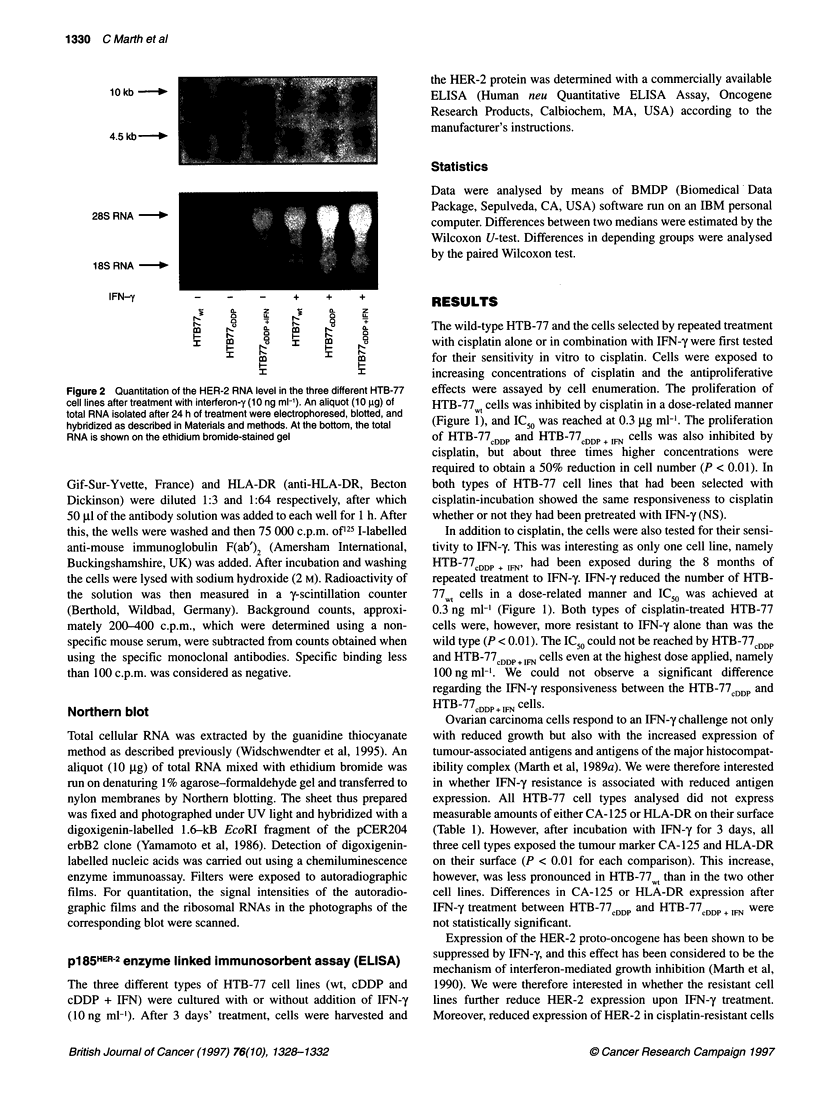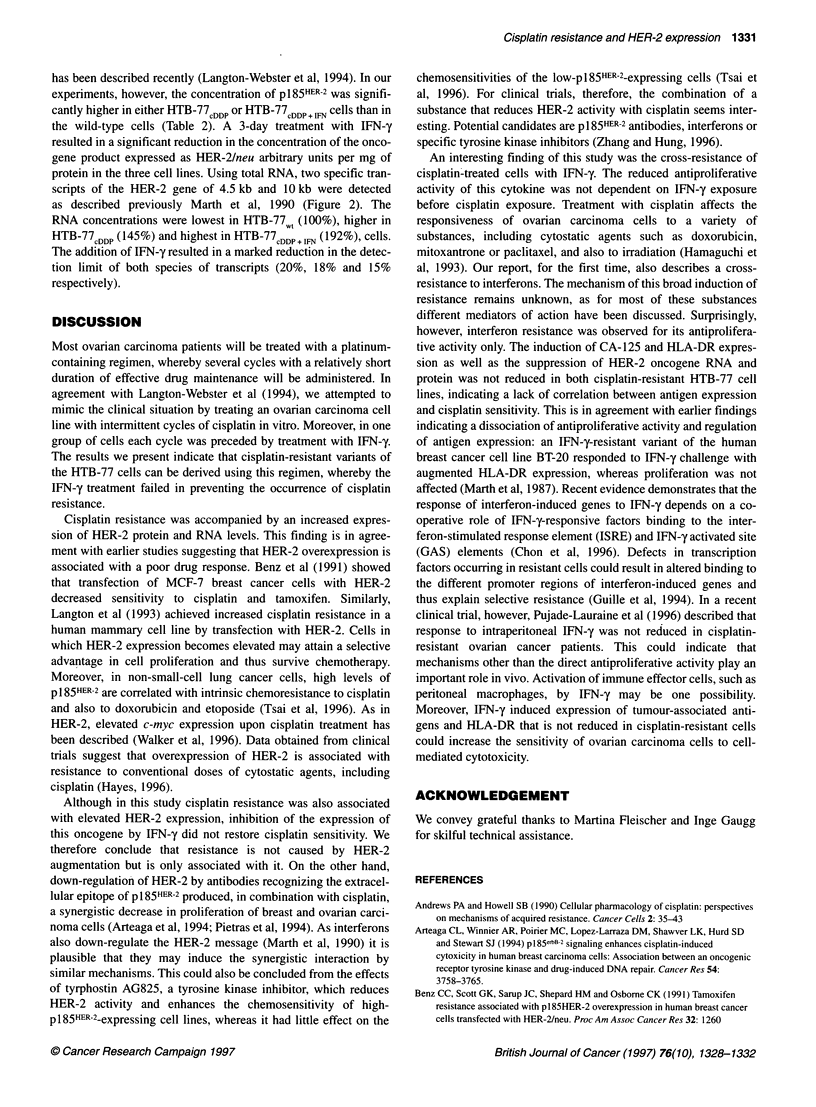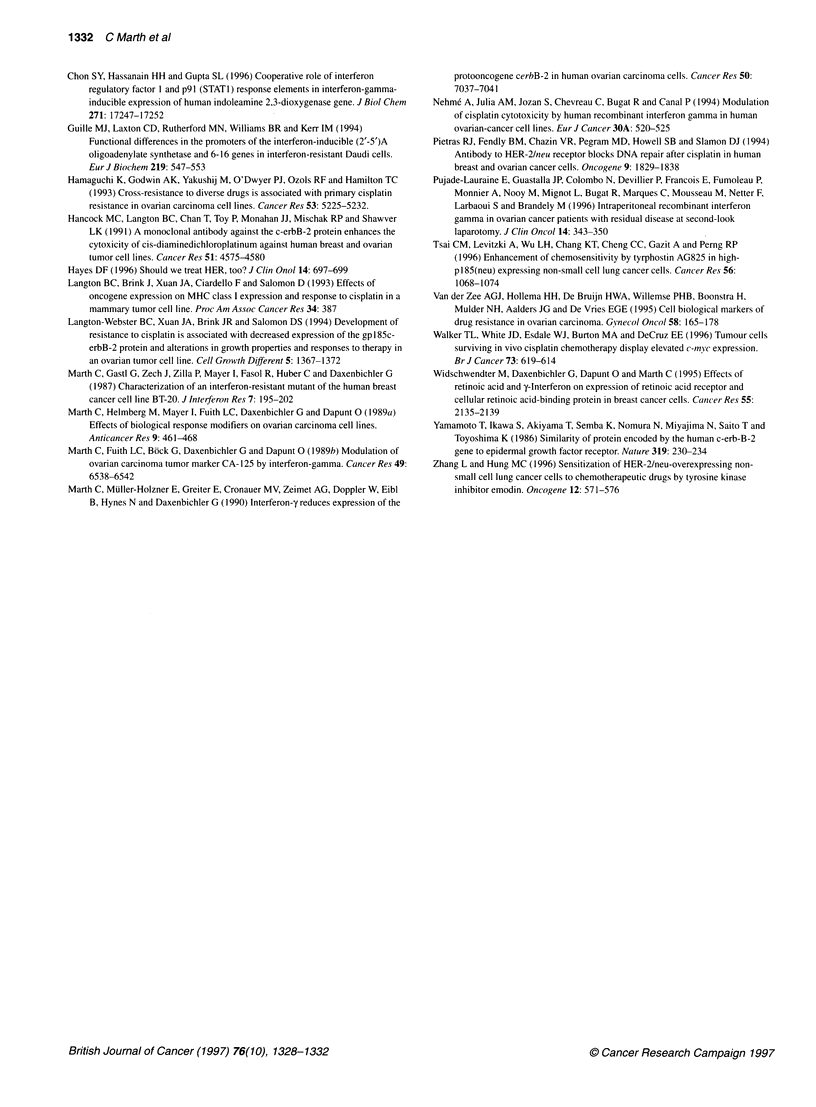# Cisplatin resistance is associated with reduced interferon-gamma-sensitivity and increased HER-2 expression in cultured ovarian carcinoma cells.

**DOI:** 10.1038/bjc.1997.556

**Published:** 1997

**Authors:** C. Marth, M. Widschwendter, J. Kaern, N. P. JÃ¸rgensen, G. Windbichler, A. G. Zeimet, C. TropÃ©, G. Daxenbichler

**Affiliations:** Department of Obstetrics and Gynaecology, University Hospital, Innsbruck, Austria.

## Abstract

**Images:**


*British Journal of Cancer* (1997) **76**(10), 1328–1332
© 1997 Cancer Research Campaign

### Cisplatin resistance is associated with reduced interferon-γ-sensitivity and increased HER-2 expression in cultured ovarian carcinoma cells

**C Marth[1,2], M Widschwendter[1], J Kærn[2], N-P Jørgensen[2], G Windbichler[1], AG Zeimet[1], C Tropé[2], and G Daxenbichler[1]**

[1]Department of Obstetrics and Gynaecology, University Hospital, Anichstrasse 35, A-6020 Innsbruck, Austria; and [2]Department of Gynecologic Oncology, The Norwegian Radiumhospital, Montebello, N-0310 Oslo, Norway

**Summary** In ovarian carcinoma cells, the combination of interferon-γ (IFN-γ) and cisplatin (cDDP) has been reported to result in a synergistic amplification of antiproliferative activity. To assess whether IFN-γ may also prevent the occurrence of cisplatin resistance, the human ovarian carcinoma cell line HTB-77 was treated repeatedly in an intermittent fashion with either cisplatin alone (HTB-$77_{cDDP}$) or cisplatin plus IFN-γ (HTB-$77_{cDDP+IFN}$). After 8 months of treatment, both new lines (HTB-$77_{cDDP}$ or HTB-$77_{cDDP+IFN}$) were found to be three times more resistant to cisplatin than the wild-type cells (HTB-$77_{wt}$). IFN-γ could not prevent the development of cisplatin resistance. Interestingly, both HTB-$77_{cDDP}$ and HTB-$77_{cDDP+IFN}$ cells were also less IFN-γ sensitive than the parental line. Both cisplatin-resistant lines expressed $p185^{HER-2}$ and HER-2 mRNA at a higher concentration than the HTB-$77_{wt}$ cells. IFN-γ was in all three HTB-77 cell lines able to suppress the HER-2 message and its encoded protein. The expression of IFN-γ-induced antigens, namely CA-125 and class II antigens of the major histocompatibility complex (HLA-DR), was markedly augmented by IFN-γ in all three lines, whereby the most prominent effect was seen in HTB-$77_{cDDP}$ and HTB-$77_{cDDP+IFN}$.

**Keywords:** cisplatin resistance; interferon; ovarian cancer; oncogene HER-2

Cisplatin (cDDP) is the most commonly used agent for the treatment of ovarian cancer. Unfortunately, the clinical use of cisplatin is limited by its toxic profile and by the frequent development of resistance (Andrews and Howell, 1990). At present, the dominant mechanism responsible for clinically acquired resistance is unclear. Possible mechanisms of resistance to platinum compounds as identified in different ovarian tumour cell lines can be divided into decreased drug accumulation, altered drug inactivation and increased repair of DNA damage (for review van der Zee et al, 1995). Modification of proto-oncogene expression has also been considered as an interesting possibility for tumour cells to overcome the toxic effect of cisplatin (Hancock et al, 1991). This has also been suggested more recently by Langton-Webster et al (1994), showing that long-term exposure of HTB-77 cells to cisplatin results in a decreased expression of the proto-oncogene HER-2 (c-*erb* B-2, *neu*), which encodes an important growth factor receptor. Interferon-γ (IFN-γ) is another drug that is able to suppress HER-2 expression in ovarian carcinoma cells, as has been demonstrated by reduced specific RNA and $p185^{HER-2}$ levels (Marth et al, 1990). As reduced HER-2 expression has been associated with increased cisplatin sensitivity, the interaction of IFN-γ with cisplatin in vitro becomes an interesting possibility for modulation of cytotoxicity: by combining IFN-γ with cisplatin we observed a synergistic effect in HTB-77 and A 2780 ovarian carcinoma cell lines and only an additive effect in OVCAR-3 ovarian carcinoma cells (Marth et al, 1989*a*). The same combination has also been shown to act in a highly synergistic manner, as demonstrated by median-effect analysis, in a cisplatin-sensitive ovarian carcinoma cell line (2008) and in its tenfold cisplatin-resistant subline (2008/C13*) (Nehmé et al, 1994). Moreover, these authors demonstrated that IFN-γ sensitized the cytotoxic effect of cisplatin.

In view of this synergistic interaction and modulation of HER-2 expression by interferons and cisplatin, we were interested to know whether IFN-γ could also prevent the development of cisplatin resistance. We selected HTB-77 ovarian carcinoma cells for their well-known responsiveness to cisplatin and IFN-γ as well as HER-2 overexpression (Marth et al, 1989*a*, 1990). By means of acute intermittent in vitro treatments with either cisplatin alone, or in combination with IFN-γ, over 8 months two HTB-77 sublines were selected and characterized for their growth properties.

### MATERIALS AND METHODS

### Substances

Recombinant DNA-derived human IFN-γ was kindly donated by Dr G Adolf, Bender, Vienna, Austria. The preparation was essentially pure and had a specific activity of $2 \times 10^7$ antiviral U per mg of protein. Cisplatin was provided by Bristol-Myers-Squibb, Vienna, Austria.

### Cell culture

The human ovarian carcinoma cell line HTB-77 (also named SK-OV-3) was obtained from Dr Christian Dittrich, University of Vienna, Austria, and was cultured and passaged in Dulbecco's modified minimum essential medium containing 10% fetal bovine serum and 2 mM L-glutamine. The HTB-$77_{cDDP}$ and HTB-$77_{cDDP+IFN}$ cells were developed by intermittent repeated exposure to either cisplatin (3 μg $ml^{-1}$) alone or a combination of

*Received 8 January 1997*
*Revised 6 May 1997*
*Accepted 12 May 1997*

*Correspondence to:* C Marth

IFN-γ (10 ng ml$^{-1}$) and cisplatin (3 μg ml$^{-1}$) respectively. Cisplatin was given to both groups simultaneously for 48 h. The interferon treatment was started in HTB-77$_{cDDP + IFN}$ cells 2 days before the cisplatin administration and continued concomitantly during the 48 h of cisplatin treatment. Surviving cells were grown to confluence (2–4 weeks) and retreated for 8 months with a total of 11 incubations. Wild-type HTB-77 (HTB-77$_{wt}$) cells were grown and passaged in parallel with two treatment groups.

### Cell proliferation

Cells were plated in 24-well tissue culture plates (Nunc, Roskilde, Denmark) and were allowed to attach overnight. The medium was changed and cisplatin or IFN-γ was added in the desired concentrations. The culture medium was renewed every 3 or 4 days. After 7 days of culture, the number of cells was enumerated by means of an electronic particle counter (Coulter, Dunstable, UK).

### Surface antigen expression

The expression of antigens belonging to the major histocompatibility complex class II (HLA-DR) or CA-125 was detected by a living cell radioimmunoassay as described previously (Marth et al, 1989*b*). Briefly, about 50 000 cells were seeded in 96-well tissue culture plates (Nunc), and after 3 days of treatment with IFN-γ (10 ng ml$^{-1}$) the wells were washed with culture medium. The mouse monoclonal antibody against CA-125 (OC-125 from CIS,

**Table 1** Effects of interferon-γ on the expression of CA-125 and HLA-DR in HTB-77 cells

| **Cell line** | **CA-125** | | **HLA-DR** | |
|---|---|---|---|---|
| | **Control** | **+ IFN-γ** | **Control** | **+ IFN-γ** |
| HTB-77$_{wt}$ | <100 | 727 ± 64 | <100 | 1973 ± 215 |
| HTB-77$_{cDDP}$ | <100 | 1921 ± 181* | <100 | 3319 ± 436* |
| HTB-77$_{cDDP + IFN}$ | <100 | 2682 ± 311* | <100 | 3652 ± 272* |

Cells were treated for 3 days with or without interferon-γ (10 ng ml$^{-1}$) and antigen expression was determined as described in Material and methods. Results are given as mean counts per min of six wells counted ± 1 s.d. *$P < 0.05$ vs HTB-77$_{wt}$ cells.

**Table 2** Effects of interferon-γ on p185$^{HER-2}$ expression

| **Cell line** | **Control** | **+ IFN-γ** |
|---|---|---|
| HTB-77$_{wt}$ | 15 400 ± 1100 | 5050 ± 550** |
| HTB-77$_{cDDP}$ | 19 200 ± 1800* | 5350 ± 480** |
| HTB-77$_{cDDP + IFN}$ | 24 000 ± 2100* | 6650 ± 510** |

Cells were cultured for 3 days with or without interferon-γ (10 ng ml$^{-1}$). Concentration of p185$^{HER-2}$ was determined as described in Materials and methods. Results are presented as mean value from six flasks measured ± 1 s.d. in HER-2/*neu* units per mg of protein. *$P < 0.05$ vs HTB-77$_{wt}$ cells; **$P < 0.05$ vs control.

**Figure 1** Effects of cisplatin (**A**) and of interferon-γ (**B**) on the proliferation of HTB-77 cells. HTB-77$_{wt}$ (●); HTB-77$_{cDDP}$ (■); and HTB-77$_{cDDP + IFN}$ (□) cells were cultured in the presence of cisplatin (**A**) or interferon-γ (**B**) for 7 days. Each point represents the mean number of cells of six wells counted in relation to the untreated control. The coefficient of variation was always below 15% and is not shown for reasons of clarity

© Cancer Research Campaign 1997

**Figure 2** Quantitation of the HER-2 RNA level in the three different HTB-77 cell lines after treatment with interferon-γ (10 ng $ml^{-1}$). An aliquot (10 μg) of total RNA isolated after 24 h of treatment were electrophoresed, blotted, and hybridized as described in Materials and methods. At the bottom, the total RNA is shown on the ethidium bromide-stained gel

Gif-Sur-Yvette, France) and HLA-DR (anti-HLA-DR, Becton Dickinson) were diluted 1:3 and 1:64 respectively, after which 50 μl of the antibody solution was added to each well for 1 h. After this, the wells were washed and then 75 000 c.p.m. of$^{125}$ I-labelled anti-mouse immunoglobulin $F(ab')_2$ (Amersham International, Buckingshamshire, UK) was added. After incubation and washing the cells were lysed with sodium hydroxide (2 M). Radioactivity of the solution was then measured in a γ-scintillation counter (Berthold, Wildbad, Germany). Background counts, approximately 200–400 c.p.m., which were determined using a non-specific mouse serum, were subtracted from counts obtained when using the specific monoclonal antibodies. Specific binding less than 100 c.p.m. was considered as negative.

### Northern blot

Total cellular RNA was extracted by the guanidine thiocyanate method as described previously (Widschwendter et al, 1995). An aliquot (10 μg) of total RNA mixed with ethidium bromide was run on denaturing 1% agarose–formaldehyde gel and transferred to nylon membranes by Northern blotting. The sheet thus prepared was fixed and photographed under UV light and hybridized with a digoxigenin-labelled 1.6–kB *Eco*RI fragment of the pCER204 erbB2 clone (Yamamoto et al, 1986). Detection of digoxigenin-labelled nucleic acids was carried out using a chemiluminescence enzyme immunoassay. Filters were exposed to autoradiographic films. For quantitation, the signal intensities of the autoradiographic films and the ribosomal RNAs in the photographs of the corresponding blot were scanned.

### p185$^{HER-2}$ enzyme linked immunosorbent assay (ELISA)

The three different types of HTB-77 cell lines (wt, cDDP and cDDP + IFN) were cultured with or without addition of IFN-γ (10 ng $ml^{-1}$). After 3 days' treatment, cells were harvested and the HER-2 protein was determined with a commercially available ELISA (Human *neu* Quantitative ELISA Assay, Oncogene Research Products, Calbiochem, MA, USA) according to the manufacturer's instructions.

### Statistics

Data were analysed by means of BMDP (Biomedical Data Package, Sepulveda, CA, USA) software run on an IBM personal computer. Differences between two medians were estimated by the Wilcoxon *U*-test. Differences in depending groups were analysed by the paired Wilcoxon test.

### RESULTS

The wild-type HTB-77 and the cells selected by repeated treatment with cisplatin alone or in combination with IFN-γ were first tested for their sensitivity in vitro to cisplatin. Cells were exposed to increasing concentrations of cisplatin and the antiproliferative effects were assayed by cell enumeration. The proliferation of $HTB\text{-}77_{wt}$ cells was inhibited by cisplatin in a dose-related manner (Figure 1), and $IC_{50}$ was reached at 0.3 μg $ml^{-1}$. The proliferation of $HTB\text{-}77_{cDDP}$ and $HTB\text{-}77_{cDDP + IFN}$ cells was also inhibited by cisplatin, but about three times higher concentrations were required to obtain a 50% reduction in cell number ($P < 0.01$). In both types of HTB-77 cell lines that had been selected with cisplatin-incubation showed the same responsiveness to cisplatin whether or not they had been pretreated with IFN-γ (NS).

In addition to cisplatin, the cells were also tested for their sensitivity to IFN-γ. This was interesting as only one cell line, namely $HTB\text{-}77_{cDDP + IFN}$, had been exposed during the 8 months of repeated treatment to IFN-γ. IFN-γ reduced the number of $HTB\text{-}77_{wt}$ cells in a dose-related manner and $IC_{50}$ was achieved at 0.3 ng $ml^{-1}$ (Figure 1). Both types of cisplatin-treated HTB-77 cells were, however, more resistant to IFN-γ alone than was the wild type ($P < 0.01$). The $IC_{50}$ could not be reached by $HTB\text{-}77_{cDDP}$ and $HTB\text{-}77_{cDDP + IFN}$ cells even at the highest dose applied, namely 100 ng $ml^{-1}$. We could not observe a significant difference regarding the IFN-γ responsiveness between the $HTB\text{-}77_{cDDP}$ and $HTB\text{-}77_{cDDP + IFN}$ cells.

Ovarian carcinoma cells respond to an IFN-γ challenge not only with reduced growth but also with the increased expression of tumour-associated antigens and antigens of the major histocompatibility complex (Marth et al, 1989*a*). We were therefore interested in whether IFN-γ resistance is associated with reduced antigen expression. All HTB-77 cell types analysed did not express measurable amounts of either CA-125 or HLA-DR on their surface (Table 1). However, after incubation with IFN-γ for 3 days, all three cell types exposed the tumour marker CA-125 and HLA-DR on their surface ($P < 0.01$ for each comparison). This increase, however, was less pronounced in $HTB\text{-}77_{wt}$ than in the two other cell lines. Differences in CA-125 or HLA-DR expression after IFN-γ treatment between $HTB\text{-}77_{cDDP}$ and $HTB\text{-}77_{cDDP + IFN}$ were not statistically significant.

Expression of the HER-2 proto-oncogene has been shown to be suppressed by IFN-γ, and this effect has been considered to be the mechanism of interferon-mediated growth inhibition (Marth et al, 1990). We were therefore interested in whether the resistant cell lines further reduce HER-2 expression upon IFN-γ treatment. Moreover, reduced expression of HER-2 in cisplatin-resistant cells

 © Cancer Research Campaign 1997

has been described recently (Langton-Webster et al, 1994). In our experiments, however, the concentration of p185$^{HER-2}$ was significantly higher in either HTB-77$_{cDDP}$ or HTB-77$_{cDDP+IFN}$ cells than in the wild-type cells (Table 2). A 3-day treatment with IFN-γ resulted in a significant reduction in the concentration of the oncogene product expressed as HER-2/*neu* arbitrary units per mg of protein in the three cell lines. Using total RNA, two specific transcripts of the HER-2 gene of 4.5 kb and 10 kb were detected as described previously Marth et al, 1990 (Figure 2). The RNA concentrations were lowest in HTB-77$_{wt}$ (100%), higher in HTB-77$_{cDDP}$ (145%) and highest in HTB-77$_{cDDP+IFN}$ (192%), cells. The addition of IFN-γ resulted in a marked reduction in the detection limit of both species of transcripts (20%, 18% and 15% respectively).

### DISCUSSION

Most ovarian carcinoma patients will be treated with a platinum-containing regimen, whereby several cycles with a relatively short duration of effective drug maintenance will be administered. In agreement with Langton-Webster et al (1994), we attempted to mimic the clinical situation by treating an ovarian carcinoma cell line with intermittent cycles of cisplatin in vitro. Moreover, in one group of cells each cycle was preceded by treatment with IFN-γ. The results we present indicate that cisplatin-resistant variants of the HTB-77 cells can be derived using this regimen, whereby the IFN-γ treatment failed in preventing the occurrence of cisplatin resistance.

Cisplatin resistance was accompanied by an increased expression of HER-2 protein and RNA levels. This finding is in agreement with earlier studies suggesting that HER-2 overexpression is associated with a poor drug response. Benz et al (1991) showed that transfection of MCF-7 breast cancer cells with HER-2 decreased sensitivity to cisplatin and tamoxifen. Similarly, Langton et al (1993) achieved increased cisplatin resistance in a human mammary cell line by transfection with HER-2. Cells in which HER-2 expression becomes elevated may attain a selective advantage in cell proliferation and thus survive chemotherapy. Moreover, in non-small-cell lung cancer cells, high levels of p185$^{HER-2}$ are correlated with intrinsic chemoresistance to cisplatin and also to doxorubicin and etoposide (Tsai et al, 1996). As in HER-2, elevated *c-myc* expression upon cisplatin treatment has been described (Walker et al, 1996). Data obtained from clinical trials suggest that overexpression of HER-2 is associated with resistance to conventional doses of cytostatic agents, including cisplatin (Hayes, 1996).

Although in this study cisplatin resistance was also associated with elevated HER-2 expression, inhibition of the expression of this oncogene by IFN-γ did not restore cisplatin sensitivity. We therefore conclude that resistance is not caused by HER-2 augmentation but is only associated with it. On the other hand, down-regulation of HER-2 by antibodies recognizing the extracellular epitope of p185$^{HER-2}$ produced, in combination with cisplatin, a synergistic decrease in proliferation of breast and ovarian carcinoma cells (Arteaga et al, 1994; Pietras et al, 1994). As interferons also down-regulate the HER-2 message (Marth et al, 1990) it is plausible that they may induce the synergistic interaction by similar mechanisms. This could also be concluded from the effects of tyrphostin AG825, a tyrosine kinase inhibitor, which reduces HER-2 activity and enhances the chemosensitivity of high-p185$^{HER-2}$-expressing cell lines, whereas it had little effect on the chemosensitivities of the low-p185$^{HER-2}$-expressing cells (Tsai et al, 1996). For clinical trials, therefore, the combination of a substance that reduces HER-2 activity with cisplatin seems interesting. Potential candidates are p185$^{HER-2}$ antibodies, interferons or specific tyrosine kinase inhibitors (Zhang and Hung, 1996).

An interesting finding of this study was the cross-resistance of cisplatin-treated cells with IFN-γ. The reduced antiproliferative activity of this cytokine was not dependent on IFN-γ exposure before cisplatin exposure. Treatment with cisplatin affects the responsiveness of ovarian carcinoma cells to a variety of substances, including cytostatic agents such as doxorubicin, mitoxantrone or paclitaxel, and also to irradiation (Hamaguchi et al, 1993). Our report, for the first time, also describes a cross-resistance to interferons. The mechanism of this broad induction of resistance remains unknown, as for most of these substances different mediators of action have been discussed. Surprisingly, however, interferon resistance was observed for its antiproliferative activity only. The induction of CA-125 and HLA-DR expression as well as the suppression of HER-2 oncogene RNA and protein was not reduced in both cisplatin-resistant HTB-77 cell lines, indicating a lack of correlation between antigen expression and cisplatin sensitivity. This is in agreement with earlier findings indicating a dissociation of antiproliferative activity and regulation of antigen expression: an IFN-γ-resistant variant of the human breast cancer cell line BT-20 responded to IFN-γ challenge with augmented HLA-DR expression, whereas proliferation was not affected (Marth et al, 1987). Recent evidence demonstrates that the response of interferon-induced genes to IFN-γ depends on a co-operative role of IFN-γ-responsive factors binding to the interferon-stimulated response element (ISRE) and IFN-γ activated site (GAS) elements (Chon et al, 1996). Defects in transcription factors occurring in resistant cells could result in altered binding to the different promoter regions of interferon-induced genes and thus explain selective resistance (Guille et al, 1994). In a recent clinical trial, however, Pujade-Lauraine et al (1996) described that response to intraperitoneal IFN-γ was not reduced in cisplatin-resistant ovarian cancer patients. This could indicate that mechanisms other than the direct antiproliferative activity play an important role in vivo. Activation of immune effector cells, such as peritoneal macrophages, by IFN-γ may be one possibility. Moreover, IFN-γ induced expression of tumour-associated antigens and HLA-DR that is not reduced in cisplatin-resistant cells could increase the sensitivity of ovarian carcinoma cells to cell-mediated cytotoxicity.

### ACKNOWLEDGEMENT

We convey grateful thanks to Martina Fleischer and Inge Gaugg for skilful technical assistance.

### REFERENCES

Andrews PA and Howell SB (1990) Cellular pharmacology of cisplatin: perspectives on mechanisms of acquired resistance. *Cancer Cells* **2**: 35–43

Arteaga CL, Winnier AR, Poirier MC, Lopez-Larraza DM, Shawver LK, Hurd SD and Stewart SJ (1994) p185$^{erbB-2}$ signaling enhances cisplatin-induced cytoxicity in human breast carcinoma cells: Association between an oncogenic receptor tyrosine kinase and drug-induced DNA repair. *Cancer Res* **54**: 3758–3765.

Benz CC, Scott GK, Sarup JC, Shepard HM and Osborne CK (1991) Tamoxifen resistance associated with p185HER-2 overexpression in human breast cancer cells transfected with HER-2/neu. *Proc Am Assoc Cancer Res* **32**: 1260

Chon SY, Hassanain HH and Gupta SL (1996) Cooperative role of interferon regulatory factor 1 and p91 (STAT1) response elements in interferon-gamma-inducible expression of human indoleamine 2,3-dioxygenase gene. *J Biol Chem* **271**: 17247–17252

Guille MJ, Laxton CD, Rutherford MN, Williams BR and Kerr IM (1994) Functional differences in the promoters of the interferon-inducible (2′-5′)A oligoadenylate synthetase and 6-16 genes in interferon-resistant Daudi cells. *Eur J Biochem* **219**: 547–553

Hamaguchi K, Godwin AK, Yakushij M, O'Dwyer PJ, Ozols RF and Hamilton TC (1993) Cross-resistance to diverse drugs is associated with primary cisplatin resistance in ovarian carcinoma cell lines. *Cancer Res* **53**: 5225–5232.

Hancock MC, Langton BC, Chan T, Toy P, Monahan JJ, Mischak RP and Shawver LK (1991) A monoclonal antibody against the c-erbB-2 protein enhances the cytoxicity of cis-diaminedichloroplatinum against human breast and ovarian tumor cell lines. *Cancer Res* **51**: 4575–4580

Hayes DF (1996) Should we treat HER, too? *J Clin Onol* **14**: 697–699

Langton BC, Brink J, Xuan JA, Ciardello F and Salomon D (1993) Effects of oncogene expression on MHC class I expression and response to cisplatin in a mammary tumor cell line. *Proc Am Assoc Cancer Res* **34**: 387

Langton-Webster BC, Xuan JA, Brink JR and Salomon DS (1994) Development of resistance to cisplatin is associated with decreased expression of the gp185c-erbB-2 protein and alterations in growth properties and responses to therapy in an ovarian tumor cell line. *Cell Growth Different* **5**: 1367–1372

Marth C, Gastl G, Zech J, Zilla P, Mayer I, Fasol R, Huber C and Daxenbichler G (1987) Characterization of an interferon-resistant mutant of the human breast cancer cell line BT-20. *J Interferon Res* **7**: 195–202

Marth C, Helmberg M, Mayer I, Fuith LC, Daxenbichler G and Dapunt O (1989*a*) Effects of biological response modifiers on ovarian carcinoma cell lines. *Anticancer Res* **9**: 461–468

Marth C, Fuith LC, Böck G, Daxenbichler G and Dapunt O (1989*b*) Modulation of ovarian carcinoma tumor marker CA-125 by interferon-gamma. *Cancer Res* **49**: 6538–6542

Marth C, Müller-Holzner E, Greiter E, Cronauer MV, Zeimet AG, Doppler W, Eibl B, Hynes N and Daxenbichler G (1990) Interferon-γ reduces expression of the protooncogene c*erb*B-2 in human ovarian carcinoma cells. *Cancer Res* **50**: 7037–7041

Nehmé A, Julia AM, Jozan S, Chevreau C, Bugat R and Canal P (1994) Modulation of cisplatin cytotoxicity by human recombinant interferon gamma in human ovarian-cancer cell lines. *Eur J Cancer* **30A**: 520–525

Pietras RJ, Fendly BM, Chazin VR, Pegram MD, Howell SB and Slamon DJ (1994) Antibody to HER-2/*neu* receptor blocks DNA repair after cisplatin in human breast and ovarian cancer cells. *Oncogene* **9**: 1829–1838

Pujade-Lauraine E, Guastalla JP, Colombo N, Devillier P, Francois E, Fumoleau P, Monnier A, Nooy M, Mignot L, Bugat R, Marques C, Mousseau M, Netter F, Larbaoui S and Brandely M (1996) Intraperitoneal recombinant interferon gamma in ovarian cancer patients with residual disease at second-look laparotomy. *J Clin Oncol* **14**: 343–350

Tsai CM, Levitzki A, Wu LH, Chang KT, Cheng CC, Gazit A and Perng RP (1996) Enhancement of chemosensitivity by tyrphostin AG825 in high-p185(neu) expressing non-small cell lung cancer cells. *Cancer Res* **56**: 1068–1074

Van der Zee AGJ, Hollema HH, De Bruijn HWA, Willemse PHB, Boonstra H, Mulder NH, Aalders JG and De Vries EGE (1995) Cell biological markers of drug resistance in ovarian carcinoma. *Gynecol Oncol* **58**: 165–178

Walker TL, White JD, Esdale WJ, Burton MA and DeCruz EE (1996) Tumour cells surviving in vivo cisplatin chemotherapy display elevated *c-myc* expression. *Br J Cancer* **73**: 619–614

Widschwendter M, Daxenbichler G, Dapunt O and Marth C (1995) Effects of retinoic acid and γ-Interferon on expression of retinoic acid receptor and cellular retinoic acid-binding protein in breast cancer cells. *Cancer Res* **55**: 2135–2139

Yamamoto T, Ikawa S, Akiyama T, Semba K, Nomura N, Miyajima N, Saito T and Toyoshima K (1986) Similarity of protein encoded by the human c-erb-B-2 gene to epidermal growth factor receptor. *Nature* **319**: 230–234

Zhang L and Hung MC (1996) Sensitization of HER-2/neu-overexpressing non-small cell lung cancer cells to chemotherapeutic drugs by tyrosine kinase inhibitor emodin. *Oncogene* **12**: 571–576

*© Cancer Research Campaign 1997*